# Does fentanyl or remifentanil provide better postoperative recovery after laparoscopic surgery? a randomized controlled trial

**DOI:** 10.1186/s12871-018-0547-z

**Published:** 2018-07-11

**Authors:** Ayako Asakura, Takahiro Mihara, Takahisa Goto

**Affiliations:** Department of Anesthesiology and Critical Care Medicine, Yokohama City University Graduate School of Medicine, 3-9, Fukuura, Kanazawa-ku, Yokohama, Japan

**Keywords:** Quality of recovery, Postoperative recovery, Opioids

## Abstract

**Background:**

Fentanyl and remifentanil are widely used opioids in surgery, but it has not been evaluated whether the choice of opioids during surgery affects the patients’ postoperative quality of recovery. Accordingly, we aim to compare postoperative recovery of fentanyl-based anesthesia with remifentanil-based anesthesia after laparoscopic surgery using the QoR 40 questionnaire (QoR-40).

**Methods:**

The study was prospective, randomized, patient and investigator-blinded, controlled, clinical trial. Seventy patients undergoing laparoscopic or retroperitoneoscopic renal or ureteral surgery were recruited and randomized to either fentanyl or remifentanil based anesthesia groups. The primary outcome was the global QoR-40 at 24 h after surgery.

**Results:**

The global median (interquartile range) QoR-40 score was 160 (138–177) in the fentanyl group (*n* = 32) and 140 (127–166) in the remifentanil group (*n* = 31). Physical comfort and physical independence, the two out of the five dimensions of the QoR-40, demonstrated significantly high scores in the fentanyl group (*P* = 0.047 and *P* = 0.032, respectively).

**Conclusion:**

Although the global QoR is higher in the fentanyl group by 20 points compared with remifentanil group, no significant differences revealed between the groups. Further studies with large numbers of subjects of the same gender are needed.

**Trial registration:**

University Hospital Medical Information Network (UMIN), UMIN000010464. Registered 10 April 2013.

## Background

The quality of recovery (QoR) after anesthesia and surgery has become an important clinical endpoint, since most patients are anesthetized safely and recover early after surgery. Quite a few studies have examined what improves postoperative QoR [1–10] and have been giving changes in our clinical practice.

Opioids with a rapid onset and short duration of action such as fentanyl and remifentanil are essential analgesics during surgery for rapid recovery. The greatest feature of remifentanil is its short context sensitive half time (3–4 min) regardless of the time of administration [11], which allows quick recovery from anesthesia despite a high plasma or effect-site concentration intraoperatively. In consequence, secretion of cortisol due to surgical stress may be suppressed too much during surgery with remifentanil-based anesthesia. As the administration of glucocorticoids before surgery improved the postoperative recovery in laparoscopic cholecystectomy [1], it’s conceivable that fentanyl-based anesthesia might provide better postoperative QoR than remifentanil-based anesthesia. However, no study examined whether the choice of opioids during surgery affects the patients’ postoperative QoR.

Therefore, the aim of the current study was to investigate whether fentanyl-based anesthesia provides better postoperative QoR than remifentanil-based anesthesia after laparoscopic surgery. The Quality of Recovery 40 (QoR-40) questionnaire, which is a global measure of postoperative recovery, was used to assess the early postoperative QoR and the 36-Item Short-Form Health Survey (SF-36 ™) was used to assess the quality of life (QoL) 1 and 3 months after surgery.

## Methods

### Participants

This study was prospective, patient and investigator-blinded, controlled, parallel-group clinical trial with equal randomization performed at the Yokohama City University Hospital. Ethical approval for this study (approval number: B120510058) was provided by the Ethics Committee of Yokohama City University Hospital (Chairperson Prof K. Ohashi), Yokohama, Japan on May 2012. The trial was registered at www.umin.ac.jp (UMIN000010464) and enrolment started from April 2013. The enrolment ended at March 2015, and the follow-up completed at July 2015.

Adult patients aged 20 to 79 years, with ASA physical status (PS) 1 and 2, who were scheduled to undergo a laparoscopic or retroperitoneoscopic renal or ureteral surgery were enrolled to the study. Patients using corticosteroid, antiemetics, opioids, or immunosuppressants; those with severe liver or renal dysfunction, poor Japanese comprehension, psychiatric disturbances, or massive blood loss during surgery; and pregnant subjects were excluded from enrolment. Written informed consent was obtained from all participants.

Subjects were randomized to either fentanyl or remifentanil based anesthesia groups, using a randomization plan with a 1:1 allocation using random block size of 10, obtained from www.randomization.com. Group assignments were sealed in sequentially numbered opaque envelopes, which were opened after the patients provided informed consent. The attending anesthesiologists were aware of the allocated arm; however, they did not take outcome measurements, and the patients, data collectors (ward nursing staffs), and data analysts were kept blinded to the allocation. In addition, all investigators, staffs, and patients were kept masked to outcome measurement and trial results.

### Perioperative management

The study subjects received no premedication. All patients received fentanyl 2 μg/kg and target controlled infusion (TCI) propofol 3–6 μg/ml for inducing anesthesia, and rocuronium 0.6 mg/kg to ease tracheal intubation. After the intubation, TCI propofol was adjusted to keep a bispectral index between 40 and 60 throughout the surgery. No volatile anesthetic was used. Rocuronium was appropriately added during the surgery to maintain the train-of-four count of 1 and 2. A catheter was placed in the radial artery to monitor blood pressure continuously and to draw blood for blood samples. In the remifentanil group, remifentanil 0.2 μg/kg/min was commenced at the induction, and was infused continuously during the surgery until the end of the insufflation. The infusing rate was controlled between 0.05 and 0.5 μg/kg/min by the attending anesthesiologist to regulate the mean arterial pressure (MAP) within 20% of preoperative values. Considering the postoperative pain after termination of remifentanil, which may lead to lower QoR-40 score, fentanyl 1 μg/kg was administered every hour, to load for postoperative patient-controlled anesthesia (PCA). In the fentanyl group, fentanyl 2 μg/kg was administered before the surgery began, and fentanyl 1 μg/kg was additionally given at any time to maintain the MAP as written above. During maintenance, F_I_O_2_ was kept between 0.4 and 0.6 and end tidal CO_2_ between 30 and 45. Core body temperature was maintained at 36–37 °C. The CO_2_ insufflation pressure was basically 10 mmHg, and was occasionally raised to 12 mmHg. At the end of the insufflation, both groups received 50 mg flurbiprofen, and PCA (CADD Legacy™, Smiths Medical Japan, Tokyo, Japan) with fentanyl (15–30 μg/ml, 1 ml/h, bolus dose 1 ml, lockout 30 min) was started. Droperidol 2.5 mg was added into PCA as PONV prophylaxis, but no other prophylaxis was administered in the operating room. No local anesthesia was administered in the ports or surgical field. Neostigmine and atropine were administered to reverse neuromuscular blocks. We confirmed that patients were not in pain before leaving the operating room. Patients were free to use non-opioid analgesics in the wards, if the pain could not be controlled by PCA.

### Data collection

The QoR-40 questionnaire [12] (Japanese version [13]) was presented to the participants 24 h after the surgical procedure. The QoR-40, as Myles introduced, “is a 40-item quality of recovery score measuring five dimensions: emotional state, physical comfort, psychological support, physical independence, and pain. Each item is rated on a 5-point Likert scale” [9], so the scores are from 40 to 200, with a higher score indicating the better QoR.

The SF-36 (Japanese version [14, 15]) was sent by mail to the subjects 1 and 3 months after surgery and was sent back by the subjects using enclosed return envelope. The SF-36 is “widely used to measure health related QoL, which consists of eight scales: physical function (PF), role limitations due to physical problems (RP), bodily pain (BP), general health (GH), vitality (VT), social functioning (SF), role limitations due to emotional problems (RE), and mental health (MH)” [16]. Scores are from 0 to 100 points, with the higher score indicating the better QoL. To minimize the loss to follow up, we sent a reminder to the subjects who had not returned the questionnaire.

Additional postoperative data collected were adrenocorticotropic hormone (ACTH), adrenaline, noradrenaline, dopamine, and cort (The samples were immediately taken to the clinical laboratory). Furthermore, data of the postoperative nausea and vomiting (PONV) and antiemetics use were collected when leaving the operating room, on 6 h and 24 h after the surgery. The pain score at rest and in motion using the numerical rating scale (NRS) from 0 (no pain) to 10 (worst pain imaginable) were asked on 6 h, 24 h, and 48 h after the surgery. The time when the patients started drinking and walking, and the total amount of bolus fentanyl consumed using the PCA were also checked.

### Statistical analysis

The primary outcome was the QoR-40 score 24 h after the conclusion of the surgical procedure. We started the study preliminary, and when 28 subjects have completed the QoR-40, we performed the power analysis to confirm eligible sample size for this study. The calculated number of patients for each group was 33 with a type 1 error of 0.05 and a power of 80%, for a difference of 13-point and standard deviation (SD) of 18.6 at this time (95% confidence interval [CI]: − 28.2 to 2.4, *P* = 0.094). We decided to recruit total of 70 subjects, considering some study participants would be lost. This was immediately reported to the Ethics Committee and was approved.

Normally distributed data are reported as the mean (± SD) and were analyzed using unpaired t test. Non-normally distributed data are reported as the median (interquartile range [IQR]) and were analyzed using Mann-Whitney test. Categorical data were compared using Fisher’s exact test. The SF-36 scores, hormones, and the pain score were compared using two-way repeated-measures analysis of variance (ANOVA) followed by between-group post hoc Student *t* tests with Bonferroni correction if significant differences revealed. Sample size analysis was performed using the R statistical software package, version 3.0.2 (R Foundation for Statistical Computing, Vienna, Austria). All other statistical analyses were performed using the GraphPad PRISM version 6.0 (La Jolla, CA), and *P*-values < 0.05 were considered statistically significant.

## Results

**Fig. 1:**
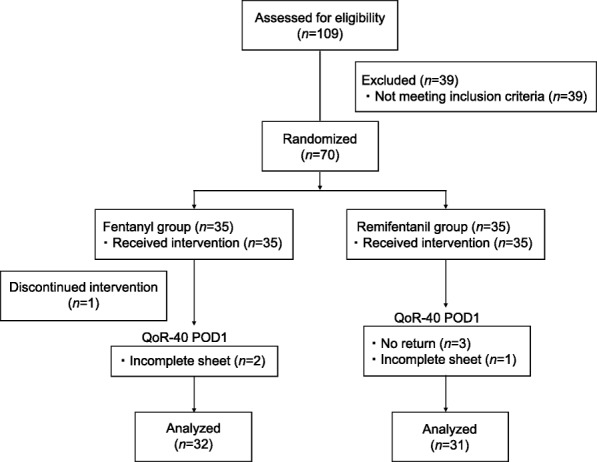
Consort flow study diagram

Seventy subjects were enrolled and randomized into treatment groups, 1 patient discontinued intervention due to the massive blood loss during the surgery; therefore, 69 subjects completed the study (Fig. 1). The number of patients analyzed for primary and secondary outcomes are shown in Fig. 1. There were no statistically significant differences between the groups in the patients’ baseline characteristics, and clinical details (Table 1). No surgical complications, readmissions, or unplanned health care contacts had occurred.

**Table 1:** Patient characteristics and clinical details

	Fentanyl (*n* = 34)	Remifentanil (*n* = 35)
Age (yr)	52.0 (39.8–66.3)	52.0 (42.0–64.0)
Sex		
M	23 (68)	24 (69)
F	11 (32)	11 (31)
Height (m)	1.66 (1.61–1.70)	1.65 (1.58–1.76)
Weight (kg)	63.1 (52.7–70.6)	67.0 (53.1–75.2)
ASA physical status		
I	13 (38)	10 (29)
II	21 (62)	25 (71)
Diagnosis		
renal carcinoma	23 (68)	27 (77)
ureteropelvic junction stenosis	11 (32)	8 (23)
Duration of anesthesia (min)	300 (267–344)	310 (260–336)
Duration of surgery (min)	217 (183–263)	230 (188–272)
Duration of insufflation (min)	168 (139–209)	166 (130–205)
Intravenous infusion (ml)	2500 ± 598	2481 ± 730
Blood loss (ml)	0 (0–75)	20 (0–50)
Total dose of fentanyl during surgery (μg)	660 (494–811)	400 (350–500)
Length of hospital stay (day)	12 (10–13)	12 (11–13)
Smoking history	12 (35)	16 (46)
Kinetosis history	4 (12)	5 (14)
PONV history	1 (3)	1 (3)

Data presented as mean ± SD, median (IQR), or number (%)

The global median (IQR) QoR-40 score presented higher values for the fentanyl group (160 [138–177]) compared with the remifentanil group (140 [127–166]), however this difference did not reach statistical significance (*P* = 0.079). Physical comfort and physical independence, the two out of the five dimensions of the QoR-40, demonstrated significantly high scores in the fentanyl group (Table 2). For the eight scales of the SF-36, GH showed a significantly high score in the fentanyl group, but no significant differences existed in the other seven scales (Table 3).

**Table 2:** Preoperative and postoperative QoR-40 dimensions and global scores

	Fentanyl	Remifentanil	*P* value
Preoperative			
emotional state	42.5 (38–45.0)	42.0 (34–45.0)	0.506
physical comfort	58.0 (54.5–60.0)	57.0 (52.0–59.0)	0.222
psychological support	35.0 (29.8–35.0)	34.0 (28.0–35.0)	0.386
physical independence	25.0 (24.5–25.0)	25.0 (24.0–25.0)	0.931
pain	35.0 (34.0–35.0)	35.0 (33.0–35.0)	0.185
global QoR-40	194.5 (177.3–198.3)	192.0 (168.0–198.0)	0.411
POD1			
emotional state	38.0 (33.3–44.0)	35.0 (30.0–39.0)	0.154
physical comfort	49.0 (44.0–55.0)	44.0 (40.0–50.0)	0.047
psychological support	29.5 (28.0–35.0)	30.0 (24.0–34.0)	0.442
physical independence	14.0 (9.0–19.0)	11.0 (8.0–13.0)	0.032
pain	28.0 (23.3–30.0)	29.0 (21.0–31.0)	0.948
global QoR-40	159.5 (138.3–177.0)	140.0 (127.0–166.0)	0.079

Data presented as median (IQR)

**Table 3:** SF-36 domain scores at 1 month and 3 months after the surgery

		Fentanyl	Remifentanil	F value	*P* value
PF	preoperative	92.7 ± 11.1	90.8 ± 16.1		
	1 month	85.2 ± 13.0	79.1 ± 20.1	F (2, 90) = 0.063	0.939
	3 months	91.1 ± 10.5	86.8 ± 20.8		
RP	preoperative	92.2 ± 20.1	90.0 ± 22.3		
	1 month	58.1 ± 24.3	56.3 ± 30.1	F (2, 90) = 0.661	0.519
	3 months	76.9 ± 25.4	84.6 ± 26.5		
BP	preoperative	78.6 ± 26.6	76.5 ± 24.0		
	1 month	56.6 ± 20.8	52.2 ± 22.8	F (2, 90) = 0.554	0.577
	3 months	78.9 ± 22.1	76.3 ± 22.4		
GH	preoperative	61.2 ± 22.8	61.0 ± 16.5		
	1 month	63.8 ± 19.3	55.3 ± 19.9	F (2, 88) = 5.191	0.007
	3 months	60.9 ± 20.7	55.4 ± 21.3		
VT	preoperative	66.8 ± 19.7	63.0 ± 21.3		
	1 month	60.4 ± 20.4	48.9 ± 23.5	F (2, 90) = 2.361	0.100
	3 months	67.1 ± 16.2	53.4 ± 24.7		
SF	preoperative	86.3 ± 17.5	86.4 ± 15.7		
	1 month	59.3 ± 26.8	57.0 ± 25.6	F (2, 90) = 0.244	0.784
	3 months	79.9 ± 26.6	76.3 ± 25.3		
RE	preoperative	89.0 ± 22.8	84.6 ± 20.6		
	1 month	72.8 ± 24.9	68.6 ± 32.1	F (2, 90) = 0.042	0.959
	3 months	78.6 ± 25.7	78.9 ± 28.6		
MH	preoperative	75.5 ± 15.0	69.2 ± 20.1		
	1 month	72.2 ± 17.0	65.1 ± 23.2	F (2, 90) = 2.863	0.062
	3 months	78.3 ± 17.4	66.4 ± 20.7		

Data presented as mean ± SD. The results of interaction (Group: Time) are shown

**Fig. 2:**
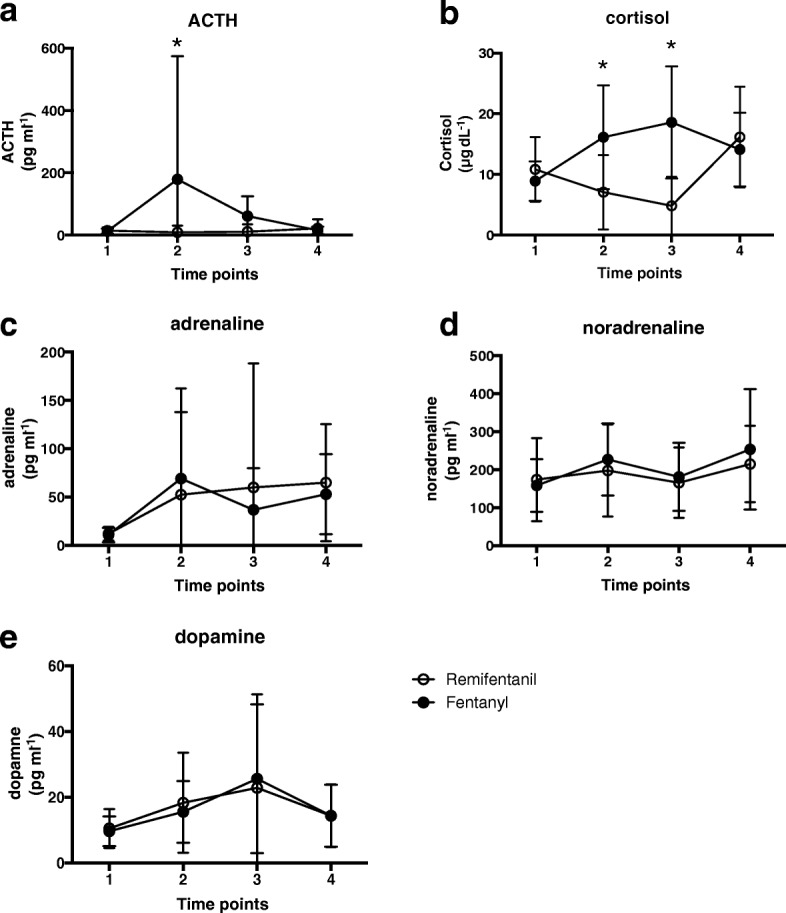
Plasma concentration of ACTH (**a**), cortisol (**b**), adrenaline (**c**), noradrenaline (**d**), and dopamine (**e**). Time points are before the surgery (1), 1 h after insufflation (2), at the end of the surgery (3), and the next morning (4). Normal ranges of ACTH, cortisol, adrenaline, noradrenaline, and dopamine were 7.2–63.3 pg ml^− 1^, 6.2–19.4 μg dl^− 1^, < 100 pg ml^− 1^, 100–450 pg ml^− 1^, and < 20 pg ml^− 1^, respectively. Data are presented as mean ± SD. * *P* < 0.01

Cortisol was significantly higher in the fentanyl group [group x time, *F*(3, 201) = 35.6, *P* < 0.001], and post hoc multiple comparisons test showed that cortisol measured 1 h after the insufflation and at the end of the surgery were significantly higher in the fentanyl group (mean difference [95% CI]: 9.1 [5.0 to 13.1], *P* < 0.001 and 13.8 [9.7 to 17.8], *P* < 0.001, respectively). ACTH was also significantly higher in the fentanyl group (*F*[3, 201] = 6.07, *P* < 0.001) and post hoc multiple comparisons test determined that ACTH measured 1 h after the insufflation was significantly higher in the fentanyl group (mean difference [95% CI]: 170.2 [84.6 to 255.8], *P* < 0.001). As for adrenaline, noradrenaline, and dopamine, no significant differences of group existed (*F*[1, 67] = 0.19, *P* = 0.66, *F*[1, 67] = 0.62, *P* = 0.43, and *F*[1, 67] = 0.01, *P* = 0.93, respectively) (Fig. 2). The incidences of nausea, vomiting and use of antiemetics assessed 6 and 24 h after surgery showed no significant differences between the groups (Table 4). The pain scores at rest and in motion on 6, 24, and 48 h after surgery, and the total dose of bolus fentanyl using the PCA demonstrated no significant differences between the groups (Table 4). Moreover, the time when the patients started drinking and walking revealed no significant differences between the groups (Table 4).

**Table 4:** Postoperative Clinical Details

		Fentanyl	Remifentanil	*P* value
Number of patients with nausea episodes	0 h	1 (2.9)	0 (0)	0.493
	6 h	3 (8.8)	2 (5.7)	0.673
	24 h	11 (32.4)	12 (34.3)	1.000
Number of patients with vomiting episodes	0 h	0 (0)	0 (0)	1.000
	6 h	1 (2.9)	1 (2.9)	1.000
	24 h	3 (8.8)	6 (17.1)	0.477
Number of patients with antiemetics use	0 h	0 (0)	1 (2.9)	1.000
	6 h	3 (8.8)	0 (0)	0.114
	24 h	5 (14.7)	5 (16.7)	1.000
Duration till drinking (h)		20 (19–23)	21 (19–22)	0.558
Pain score in motion	6 h	3.2 ± 2.7	4.4 ± 2.2	0.338
	24 h	4.0 ± 2.3	4.9 ± 2.3	
	48 h	4.2 ± 2.7	4.2 ± 2.1	
Pain score at rest	6 h	1.6 ± 2.0	2.1 ± 2.0	0.337
	24 h	1.6 ± 1.8	2.1 ± 2.0	
	48 h	1.3 ± 1.7	1.4 ± 1.2	
Duration till walking (h)		25 (22–44)	42 (22–45)	0.736
Total dose of bolus fentanyl used in PCA (μg)		25 (0–184)	30 (0–150)	0.978
Use of non-opioid analgesics		4 (11.8)	6 (17.1)	0.734

Data presented as mean ± SD, median (IQR), or number (%)

Pain score were analyzed using two-way ANOVA. The results of group difference are shown

## Discussion

We have identified no significant differences in global QoR-40 score 24 h after surgery, between the fentanyl and the remifentanil groups. However, within the five dimensions, physical independence scores were significantly higher in the fentanyl group. Cortisol and ACTH measured during and at the end of the surgery showed significantly high plasma concentration values in the fentanyl group. No differences were observed in the incidence of PONV, the pain score, postoperative fentanyl consumption, and the time when patients started drinking or walking.

The fentanyl group showed a higher global QoR-40 score by 20 points than the remifentanil group, which is more than three times higher of minimal clinically important difference (MCID) for the QoR-40 that has been reported as 6.3 [17]. Therefore, the difference between the groups is largely relevant. Nevertheless, no statistically significance was apparent. Two possible explanations for the results could be considered. First, despite the need for at least 66 subjects, primary outcome data were available for only 63 subjects. Three patients had some questions skipped, and 3 patients had lost the questionnaire at their home (Fig. 1), which were unfortunately, more than we have expected. Second, the overall SD was 18 when we performed the sample size calculations with 28 subjects; however, it was 22 when the final analyses were performed with 63 subjects. Therefore, the allocation bias seems to be not minimized by the randomization. Overall, we could state that the current study was underpowered. In addition to these, we should discuss whether the MCID derived from the Australian study [17] could directly extrapolate to the Japanese population. The SD of the QoR40 score in the validation study of the Japanese version was also 22 [13], which was 1.6 times higher than that of the Australian study (i.e. 14) [17]. Because in general, the MCID become higher with a higher SD, the MCID in Japanese population could be 1.6 times higher (i.e. 10) than the Australian population. Nevertheless, the difference of the QoR40 in our study still exceeded the MCID.

There was a significant difference in the physical comfort dimension. This dimension asks about breathing, sleeping, eating, resting, PONV, shivering, etc. As there were no differences in the PONV and the time of the patients’ drinking, the significant difference must have occurred in one of the other remaining factors. There also was a significant difference in physical independence. Although the time when the patients started walking was not significantly different, their medians were 25.5 and 42.0 for the fentanyl and remifentanil groups, respectively. This is almost a day different and could have affected the score in the physical independence dimension, which was assessed on POD1.

With a longer period of time to recover from anesthesia and surgery, it is intriguing that the significant difference appeared in the GH domain of the SF-36. This domain mainly questions about whether the subjects are feeling healthy or not. Although it might not be related much to the time course of recovery, fentanyl might have a better effect on QoL a few months after surgery, compared with remifentanil.

The plasma concentration levels of cortisol and ACTH during surgery were higher in the fentanyl group, which is consistent with past study that remifentanil suppressed the increase in ACTH and cortisol during laparoscopic colectomy compared with epidural anesthesia [18]. The global QoR-40 score tended to be higher in the fentanyl group, nevertheless, no correlation between the global QoR-40 score and cortisol was apparent (*r* = 0.089). In the previous studies, premedication with ibuprofen improved the QoR-40 of POD1 in spite of intraoperative cortisol levels as high as control group [8]. Furthermore, intraoperative infusion of dexmedetomidine showed no difference in the QoR-40 score on POD1, although cortisol levels were significantly lower than control group after surgery [7]. Altogether, intraoperative cortisol seems not relevant to the QoR-40 score on POD1. Multiple factors are responsible for recovery from surgery, and thus we considered that some other factors beside hormone have affected the QoR-40 score, which, disappointingly, cannot be clarified in our study design.

There are limitations to our study. First, as previously discussed in detail, the study was underpowered. Second, the SF-36 is not specifically designed for use after surgery, so it may not be reliable for measuring the intermediate to the late phase postoperative recovery. Perhaps, we should have used other tools [19], e.g. the functional recovery index [20], the surgical recovery index [21], or the postoperative quality of recovery score [22]. Third, small amount of fentanyl was used periodically throughout the management of remifentanil group, to base fentanyl for postoperative PCA. However, we considered that not using fentanyl might lead to lower QoR-40 score with stronger postoperative pain in remifentanil group. Owing to this reason, minimum amount of fentanyl was given in the remifentanil group. Fourth, external validity is low, and our results may not apply to patients with severe comorbidities or patients undergoing more invasive surgery, since the enrolled subjects were relatively healthy patients who underwent less invasive surgery. In addition, two thirds of the participants were male. Patient sex is known to affect QoR [23], thus, we should have recruited patients of either sex.

## Conclusion

In conclusion, although the global QoR is higher in the fentanyl group by 20 points compared with remifentanil group, the current study could not reveal significant differences in global QoR between fentanyl and remifentanil groups. Further randomized controlled trials with large number of subjects of the same gender are needed to assess whether fentanyl or remifentanil provide better postoperative QoR.

## Abbreviations

ACTH: Adrenocorticotropic hormone
ANOVA: Analysis of variance
BP: Bodily pain
CI: Confidence interval
GH: General health
IQR: Interquartile range
MAP: Mean arterial pressure
MCID: Minimal clinically important difference
MH: Mental health
NRS: Numerical rating scale
PCA: Patient-controlled anesthesia
PF: Physical function
POD: Postoperative day
PONV: Postoperative nausea and vomiting
PS: Physical status
QoL: Quality of life
QoR: Quality of recovery
RE: Role limitations due to emotional problems
RP: Role limitations due to physical problems
SD: Standard deviation
SF: Social functioning
SF-36: 36-Item Short-Form Health Survey
TCI: Target controlled infusion
VT: Vitality
